# A Metabolomic Perspective on Coeliac Disease

**DOI:** 10.1155/2014/756138

**Published:** 2014-02-09

**Authors:** Antonio Calabrò, Ewa Gralka, Claudio Luchinat, Edoardo Saccenti, Leonardo Tenori

**Affiliations:** ^1^Department of Experimental and Clinical Biomedical Sciences, University of Florence, Viale Pieraccini 6, 50139 Florence, Italy; ^2^Tuscany Referral Center for Adult Coeliac Disease, Viale Pieraccini 6, 50139 Florence, Italy; ^3^Magnetic Resonance Center (CERM), University of Florence, Via L. Sacconi 6, 50019 Sesto Fiorentino, Italy; ^4^Department of Chemistry, University of Florence, Via della Lastruccia 3, 50019 Sesto Fiorentino, Italy; ^5^Biosystems Data Analysis Group, University of Amsterdam, 904 Science Park, 1098 XH Amsterdam, The Netherlands; ^6^Laboratory of Systems and Synthetic Biology, Wageningen University and Research Center, Dreijenplein 10, 6703 HB Wageningen, The Netherlands; ^7^FiorGen Foundation, Via L. Sacconi 6, 50019 Sesto Fiorentino, Italy

## Abstract

Metabolomics is an “omic” science that is now emerging with the purpose of elaborating a comprehensive analysis of the metabolome, which is the complete set of metabolites (i.e., small molecules intermediates) in an organism, tissue, cell, or biofluid. In the past decade, metabolomics has already proved to be useful for the characterization of several pathological conditions and offers promises as a clinical tool. A metabolomics investigation of coeliac disease (CD) revealed that a metabolic fingerprint for CD can be defined, which accounts for three different but complementary components: malabsorption, energy metabolism, and alterations in gut microflora and/or intestinal permeability. In this review, we will discuss the major advancements in metabolomics of CD, in particular with respect to the role of gut microbiome and energy metabolism.

## 1. Introduction

Coeliac disease (CD, MIM 212750), first described in 1887, is a common complex chronic immune-mediated disorder with a known (gluten) environmental trigger. Recent surveys indicate that it may affect 1 in 105 subjects in the United States [1], 1 in 67 Finnish school children [2], and 1 in 230 in Italian school age children [3], with seroprevalence of about 1% in subjects of white European origin [4, 5].

Coeliac disease has a strong genetic component with multiple contributing genes: the most important and best characterized genetic risk factors are the HLA class II genes DQ2 and/or DQ8 which are located on chromosome 6p21. More than 97% of patients have at least one of the two genes: most patients (>90%) carry the DQ2 gene, while the rest expresses the DQ8 gene. HLA-DQ2 is encoded by the HLA-DQA1*05 allele (*α* chain) and the HLA-DQB1*02 (*β* chain) [6, 7]. Common to many other autoimmune disorders, the two alleles are often present in the *cis* conformation on the DR3 haplotype [8]; HLA-DQ2 and HLA-DQ8 are necessary but not sufficient for the development of CD.

Genome wide association studies indicated 39 non-HLA loci to be predisposing to CD [9–11]. Altogether, the nonHLA loci explain only 5% of the risk for CD [6], while the HLA loci account for 35% of the risk [7].

Several of these nonHLA CD susceptibility genes are associated with other diseases/traits [6] such as type 1 diabetes [12, 13], rheumatoid arthritis [14], and systemic lupus erythematosus [15] indicating a possibly shared genetic background with other diseases [7].

The environmental trigger of coeliac disease is gluten, a protein complex formed by gliadin and glutenin, which is found in wheat and related grain species like barley and rye and also in processed food where it is used to enhance food texture and as a stabilizing agent.

The active disease component is gliadin [16] with the *α*-,  *β*-,  *γ*-, and *ω*-fractions. These fractions are rich in proline and glutamine and resistant to enzymatic digestion; large proline/glutamine-rich peptides accumulate in the smallest intestine, triggering an abnormal innate and adaptive immune response in genetically predisposed subjects.

The response of the adaptive immune system is due to the gliadin-reactive CD4+ T cells; HLA-DQ molecules bind to these peptides which are deamidated by the intestinal brush border enzyme tissue transglutaminase; these complexes interact with the T-cell receptor on T cells leading to T-cell activation with subsequent release of proinflammatory cytokines and the production by B-cells of specific antibodies (anti-tissue transglutaminase and endomysial antibodies) [17, 18].

The role of the innate immune systems in CD is less clear [7]. Increased expression of interleukin-15 has been observed [19]; enhanced intestinal permeability has been also observed [20], induced by zonulin [21], whose release is mediated by gluten activated CXCR-3 [22, 23]. Loss of functionality in the intestinal barrier permits the passage of immunoreactive peptides and other antigens from the gut lumen to the lamina propria, with subsequent triggering of the innate immune system.

## 2. Metabolomics

The advent of high-throughput techniques led to a rapid expansion of data sets originated from the analysis of gut microbiota and currently several ongoing projects are aimed at the study and definition of the microbiome [24, 25]. In this framework, metabolomics is playing a crucial role.

Since the systematic genome sequencing of the first free-living microbe [26], we have seen the rising of genome-wide expression profiling methods, aimed to understand complex biological systems on a large scale [27]. The fast development of genomics, transcriptomics, proteomics, and the other *omics* disciplines is the consequence of this new scientific paradigm.

In this framework, metabolomics has already proved in the past decade to be a useful complement for the characterization of several physiological and pathological conditions and offers promises as a clinical tool [28]. Metabolomics is based on the analysis of the measured dynamic changes of a living organism in response to genetic modifications or physiological stimuli such as nutrients, drugs treatment, or toxic insults [29].

**Figure 1:**
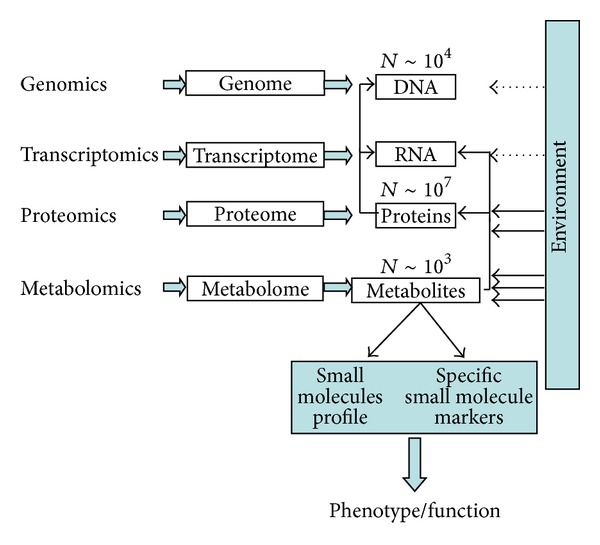
Relationships between the omics sciences.

The metabolome, the complete collection of all metabolites contained in a biological specimen, can be considered the downstream end-product of the complex interaction of genome, transcriptome, proteome, and the environment: it can be regarded as a cascade linking genome to the phenotype [30] (Figure 1). The metabolome, consisting of low-molecular weight chemical intermediates [31], can be considered as an amplified version of gene expression. While changes in gene expression levels (and thus proteins) will have only small effects on metabolic fluxes, they must have large effects on metabolic pathways (and thus metabolites concentrations) [27]. From this point of view, the metabolite space represents the optimal level at which changes in biological systems are analyzed with optimal sensitivity [32] under conditions of negligible effects on the global phenotype [33].

Metabolomics does not rely on the measurement of a single metabolite but considers the spectrum of (possibly) all metabolites as a whole, taking a holistic approach; this offers evident advantages with respect to a targeted search of metabolites; indeed, no assumption is required on the identity of the metabolites that are or may be relevant for the biological phenomenon under investigation.

The main analytical techniques employed in metabolomics (Boxes 1 and 2) are nuclear magnetic resonance spectroscopy (NMR) and mass spectrometry (MS). Both MS and NMR methods provide information on the relative and absolute concentrations of different classes of metabolites in a single measurement (see Box 3) and can be also used to determine metabolite structures providing mechanistic insights.

**Figure 2:**
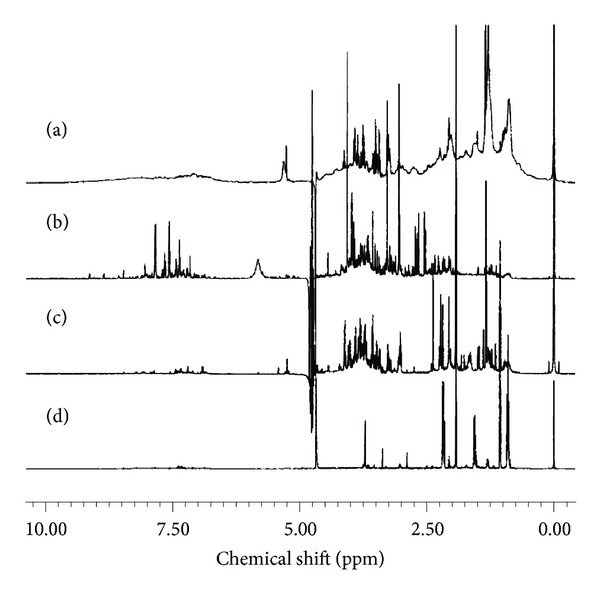
Examples of NMR profiles of (a) serum, (b) urine, (c) saliva, and (d) faecal extract.

The most common biological specimens used in metabolomics are serum/plasma and/or urines, firstly, because they can be collected with low invasiveness, and, secondly because, as they contain thousands of metabolites, they are rich in biological information at the systemic level; a number of other biofluids such as saliva [34], tissue extract [35], cerebrospinal fluid [36], bile [37], seminal fluid [38], amniotic fluid [39], synovial fluid [40], exhaled breath condensate [41, 42], and faecal extracts [43] can also be studied.  Figure 2 shows typical NMR spectra of four different biofluids (Box 3).

Targeted and untargeted approaches are possible in metabolomics, the former focusing on the analysis of a subset of known compounds or class thereof (targeted MS and NMR) and the latter focusing on the whole array of metabolites within the detection limit of the technique employed (untargeted MS and NMR). Using both approaches, hundreds to thousands of metabolites are measured. Data are usually analyzed following the classical metabolomics pipeline (Box 4), and information is extracted using state-of-the-art statistical tools (Box 5).

Metabolomics has provided significant information on a wide range of pathologies, such as cancer [44], meningitis [45], neurological disorders [46], cardiovascular diseases [47], inborn errors of metabolism [48], and CD [49–51]. The first metabolomics investigation of CD revealed that a metabolic fingerprint for coeliac disease can be defined [49], which accounts for three different but complementary components: malabsorption, energy metabolism, and alterations in gut microflora and/or intestinal permeability.

In this review, we will discuss the major advancements in metabolomics of CD with respect to the role of gut microbiome and energy metabolism.

## 3. Gut Microbiota and CD

Recent studies [52–56] pointed to the possible role of intestinal microbiota (faecal and duodenal species) in the development of coeliac disease. A summary of the most relevant findings in this research area is reported in Table 1 together with the associated bacteria strains involved.

Nistal et al. [57] compared the differences between gut microorganisms in the upper small intestinal mucosa in adults and in children. A two-time higher number of microbial genera have been identified in adults compared to children, although the dominant genera were very similar: *Firmicutes*, *Proteobacteria*, *Bacteroidetes*, *Actinobacteria,* and *Fusobacteria*. Differences in the amount of bacterial communities between adult and juvenile groups can be also directly connected with the age of investigated subjects.

A comparison between healthy, diseased, and treated coeliac adults showed a lower number of *Streptococcus* spp. and *Prevotella* spp. families in untreated coeliac adults. Interestingly, similar patterns were also observed in CD children, suggesting that these bacterial populations may have been modified by changes in the intestine environment caused by active CD.

In the study by Nadal et al. [58], the bacterial species present in faeces and duodenum of children with active and treated CD were compared with a healthy control group. The ratio of harmless Gram-positive bacteria (*Lactobacillus* + *Bifidobacterium*) to potentially harmful Gram-negative (*Bacteroides*/*Prevotella* + *E. coli*) bacteria was significantly lower in CD patients than in controls, while no distinction was possible between active and inactive CD.

Sánchez et al. [59] applied denaturing gradient gel electrophoresis (DGGE) to analyze intestinal microbiota from biopsy specimens obtained from three groups of children, investigating the composition of *Bacteroides*, *Bifidobacterium*, and Lactic acid bacteria in duodenal biopsies of patients with active and treated coeliac disease. Dysbiosis in *Bacteroides* (the most abundant intestinal bacterial group) was observed, with a significant reduction in coeliac and coeliac treated patients in comparison with the control group. Moreover, it was observed that a treatment with gluten-free diet did not restore the balance of the* Bacteroides* composition. Interestingly, it was observed that the lactic acid bacteria (*Lactobacillus*) and the *Weissella* family were more abundant and diverse in treated coeliac and control patients than in patients with untreated coeliac disease. The authors suggested that some of the changes in duodenal bacterial community could be due to the inflammatory consequences of the active phase of the disease; nonetheless, the influence of different dietary habits could not be discarded.

Biopsies from treated coeliac children were analyzed in a study by di Cagno et al. [63], that also confirmed that a gluten-free diet lasting two or more years is not able to restore completely the microbiota. In addition, a higher diversity of the *Eubacteria* community was observed in the duodenum of coeliac children under a gluten-free diet with respect to healthy children. Compared to that of duodenal biopsies, the faecal bacterial population was found to be more diverse. PCR-DGGE faecal profiles of *Lactobacillus* and *Bifidobacterium* differ between treated coeliac children and healthy controls. The ratio between *Lactobacillus*/*Bifidobacterium* and *Bacteroides*/*Enterobacteria* was lower in coeliac children under treatment compared to healthy children. Some of the differences could be related to both coeliac disease and dietary variations.

Surprisingly, a study by Ou et al. [65], based on biopsies collected from the distal duodenum/proximal jejunum of 45 children, did not reveal significant differences between the microbiota in the small intestine of diseased and healthy children, although bacteria from the *Haemophilus* family were more abundant in CD patients, while *Neisseria polysaccharea* were more widespread in the control group. However, at the *genus* level, no differences between the two groups were observed. The authors commented that differences at the species level could not be excluded because complete 16 S rDNA were not sequenced. Similar results, pointing to a lack of significant differences in global composition of duodenal microbiota between healthy controls and CD patients, were obtained also by Cheng et al.; on the other hand, a subpopulation profile, containing eight genus-like bacterial groups, was found to distinguish healthy controls from CD patients [61].

The possible effect on microbiota of different types of milk feeding in early life and the link to the risk of CD development were investigated by Sánchez et al. [60]. The study was carried out on stools of breast-fed infants with different genetic risk of CD (low and high); it showed that high-risk infants had a higher prevalence of *Bacteroides vulgatus*, whereas low-risk infants had higher population of *B. uniformis*, *B. ovatus*, and* B. plebeius*. In the study group of formula-fed infants, *B. ovatus* and *B. plebeius* were increased in subjects with lower genetic risk, while *B. vulgatus* had higher prevalence in those subjects with higher genetic risk. The authors concluded that both types of milk feeding in conjunction with HLA-DQ genotype can influence the *Bacteroides* colonization, increasing the risk of coeliac disease onset. Also, the time of exposure to milk feeding was found to be relevant in prompting coeliac disease development [66].

Sellitto et al. [51] reported the impact on the intestinal tract of two different patterns of gluten introduction. A delay in gluten exposure of at least 6 to 12 months was found to have a positive effect on gluten tolerance: it caused a delay in CD autoimmunity onset in infants that were genetically susceptible to CD. Instead, the early exposure to gluten of infants at risk of coeliac disease was found to induce an immune response and led to a more frequent development of CD. Moreover, a lack of gut microflora maturation during the first 2 years of life in infants at risk of CD was also noted. The gut metabolome of the first 6 months of infant's life reflects mainly the milk diet (rich in polysaccharides and other sugars) and is very similar in all infants; once the solid food is introduced, a shift occurs and a group of short-chain fatty acids are found in faeces. By the end of the second year of life, *Bacteroides* are the main bacteria group found in the metabolome of healthy infants. Conversely, in infants with a genetic risk of celiac disease, an overall lack of bacteria of the phylum *Bacteroides* and abundance of *Firmicutes* were observed.

Recent studies [67] suggest that the colonization of gastrointestinal tract is very important in the development of autoimmune disorders and food-related disease. Furthermore, possible interaction between the intestinal bacteria and the mammalian immune system in the direct differentiation of both pro- and anti-inflammatory T-cells population has been suggested [68]. To clarify whether the gut microflora present in the faeces of CD patients is involved in the proinflammatory activity of coeliac disease, *Bifidobacterium* from healthy subjects was co-incubated together with the faecal microflora or the peripheral blood mononuclear cell culture of coeliac subjects [64]. It appeared that certain strains of *Bifidobacterium* are able to suppress and reverse the proinflammatory effect by increasing IL-10 cytokine production. These results may suggest the use of selected strains of *Bifidobacterium* as probiotics for treatment of CD.

It has been also suggested that gluten intolerance may be also triggered by environmental factors like viruses or bacteria showing molecular mimicking with gluten proteins, causing an autoimmune response that may last even after infection [69]. Several studies pointed to infections by human adenovirus [70], hepatitis C virus [71], rotaviruses [72], or *Campylobacter jejuni* [73] that could induce allergic reactions similar to that induced by gluten exposure, causing the onset of CD.

## 4. Body Composition and Energy Expenditure in CD Patients

Patients with the classic form of coeliac disease are always characterized by weight loss directly connected with malabsorption and subsequent risk of malnutrition. Often coeliac disease results in a general lack of energy and strength that can create abnormal conditions described as (chronic) fatigue. Appearance of fatigue and fatigue-related problems seems to be more frequent in nontreated coeliac patients than in patients on a gluten-free diet [74].

Body composition, resting metabolic rate (RMR), and substrate oxidation rates were investigated in [75, 76]. The results showed that untreated and treated CD patients had a lower body weight, lower levels of fat-free mass (FFM), and lower fat mass (FM) in comparison to the healthy controls. In [77], the analysis of body composition at the diagnosis time and after one year of treatment with a gluten-free diet was carried out. The analysis showed a significant increase of body weight and FM but only a slight increase of FFM after treatment with gluten-free diet. Additionally, RMR values were higher in CD patients (treated and untreated) than in controls. Moreover, untreated CD patients showed a higher npRQ (nonprotein respiratory quotient); this may indicate that untreated patients oxidize larger amounts of carbohydrate under resting metabolite conditions than treated CD and healthy subjects.

Interestingly ghrelin, one of the hormones responsible for energy balance regulation, is also changed in CD patients. Ghrelin is a 28-amino acid-peptide produced by the enteroendocrine cells of the gastric mucosa and the intestine [78]. Recent studies have shown that ghrelin is able to increase food intake, decrease fat use, and reduce energy expenditure [79]. While serum ghrelin concentration was increased in CD patients, body mass was decreased [80, 81]. Lower levels of circulating ghrelin were found in CD patients after gluten-free treatment in comparison with CD and control subjects [82]. These results suggest that low amounts of ghrelin in the blood may be partially responsible for the slight increase in body weight and FM in CD patients after treatment with a gluten-free diet.

## 5. Metabolomic Signature of CD

In many cases, the diagnosis of CD is not an easy task, mainly because CD has a variable clinical picture due to its intertwingled genetic, immunological, and environmental components. The presence of the HLA genetic factor, together with a positive biopsy and serological antibodies upon gluten-containing diet, is used to diagnose coeliac disease at any age. In order to better understand the processes underlying the activation and development of coeliac disease, it is important to examine the mechanisms from the early beginning.

To date, a limited number of metabolomics studies of coeliac disease are available, but they clearly show that metabolic differences between healthy individuals and coeliac patients exist. In the first (to our knowledge) metabolomic study on CD, Bertini et al. [49], examined adult healthy controls and coeliac patients by ^1^H NMR profiling of their serum and urine profiles before and after GFD, showing that a metabolic fingerprint for CD can be defined. This fingerprint was found to be made up by three components, one related to malabsorption, one related to energy metabolism, and the third related to alterations in gut microflora and/or intestinal permeability. Using this metabolic fingerprint, it was possible to make predictions about the coeliac status with a very good accuracy (ca. 84%). One of the most interesting findings was that the metabolic profile of CD patients reverts to normality after 12 months of a strict gluten-free diet; interestingly, a similar behavior was not found in CD patients when analyzing them from a gut microflora prospective [58, 59, 63].

The main observed differences in serum spectra between CD patients and controls were lower levels of several amino acids (asparagine, isoleucine, methionine, proline, and valine), methylamine, pyruvate, creatinine, choline, methylglutarate, lactate, lipids, and glycoproteins and higher levels of glucose and 3-hydroxybutyric acid. Notably, the best discrimination is obtained from CPMG spectra (Carr-Purcell-Meiboom-Gill spin echo sequence) [83], that is, from spectra in which signals arising from large macromolecules such as lipidic components are suppressed [49]. So, although it is known that coeliac patients usually appear to be hypocholesterolemic, lipids do not contribute significantly to the metabonomic signature of coeliac disease. A decrease in the level of pyruvate and lactate and a higher level of glucose in the blood of coeliac patients were observed, probably as a consequence of an impaired glycolysis process. Glycolysis impairment can cause a lowering of pyruvate and lactate levels and an increase of glucose levels in blood. If this metabolic way is reduced, *β*-oxidation is probably increased. Enhanced *β*-oxidation and malabsorption can then explain lower levels of lipids in serum [49]. In these conditions, the authors hypothesized an increase of the use of ketonic bodies as a source of energy in coeliac patients, consistently with the higher observed levels of 3-hydroxybutyric acid in blood and acetoacetate in urines [49].

Energy conversion from lipids and catabolism of ketonic bodies are far less efficient than that from glucids. Untreated coeliac subjects often report symptoms of fatigue. In patients on a gluten-free diet, fatigue tends to be reduced and, in fact, it has been proposed that this condition is gluten-related [74]. In [49], the authors found that in CD patients on a gluten-free diet the levels of glucose and 3-hydroxy-butyric acids revert to normality.

Further, the authors found that CD patients are characterized by higher urine levels of some metabolites related to gut microbiota: indoxyl sulfate (IS), meta-[hydroxyphenyl] propionic acid (m-HPPA), and phenylacetylglycine (PAG). M-HPPA mostly originates from gut microflora, being one of the several products of the microbially mediated breakdown of larger plant phenolic compounds such as caffeic acid and its conjugate chlorogenic acids [84]. IS is a harmful uremic toxin produced in the liver from indole through indoxyl. Indole is a subproduct of tryptophan metabolism by intestinal bacteria [85]. Modulation of PAG excretion in urine has been attributed to gut microflora, and increases of PAG have been reported in cases of drug-induced phospholipidosis; nonetheless, the contribution of mammalian and microbial sources to PAG excretion is not yet fully characterized [86]. All these findings are consistent with the hypothesis that in CD patients the gut microflora of the small bowel is altered or presents peculiar species with their own microbial metabolome.

In a following investigation [50], the same research group highlighted again the existence of a metabolic fingerprint for coeliac disease, confirming most of the previously discussed metabolites with the additional finding of higher levels of *p*-cresolsulfate in the urines of CD patients. Interestingly, *p*-cresolsulfate, a metabolite of bacterial origin, is associated with several gastric-related disease [87], including bowel cancer [88]. In the same study, the analysis of the so-called “potential coeliac patients” (i.e., subjects who have a positive antibody test but no evidence of intestinal damage) showed that the metabolic patterns of overt and potential coeliac patients are similar [50] indicating that CD-related dysmetabolism precedes the intestinal damage. Only a few serum metabolites differentiate between potential and overt CD, and none of these metabolites are related to the energy metabolism [50]. It appears that, as in overt CD patients, glycolysis is somehow impaired also in potential CD patients. Impairment of glycolysis explains both the observed lower lactate levels and the higher glucose levels in blood of potential CD patients. In urine, there are more metabolites that discriminate potential and overt CD. The key differences lie in the concentration of metabolites originating from the gut microflora (m-HPPA, IS, and PAG) which in potential coeliac subjects are similar to those of controls, suggesting a relationship between overt CD, villous atrophy, and bacterial consortia of the host [50].

**Figure 3:**
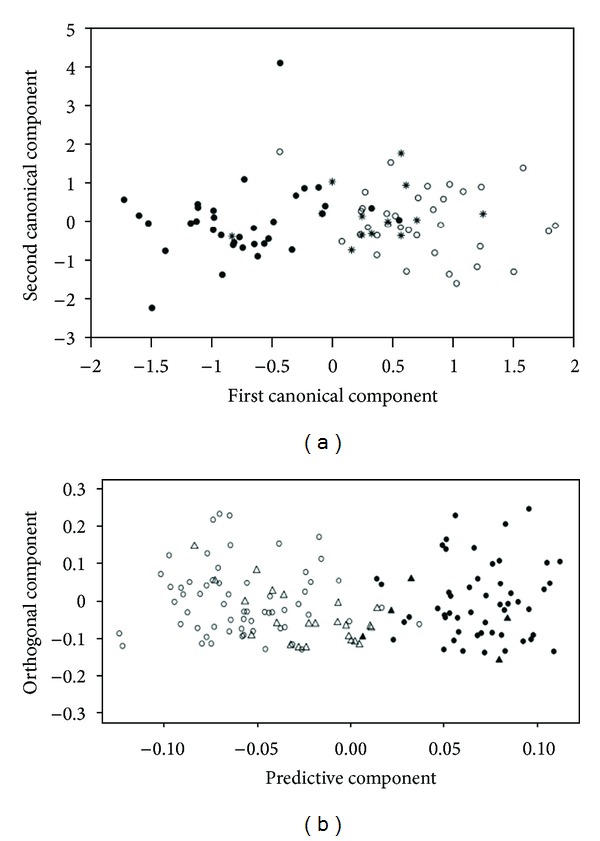
(a) Clustering of CMPG (Carr-Purcell-Meiboom-Gill spin echo sequence) [83] serum spectra of CD patients (filled circles) and controls (open circles). The discriminant model between the two groups was calculated using a combination of partial least square [89] and (regularized) canonical analysis [90] (PLS-RCC) and was validated using cross-validation. The CPMG spectra of 13 (out of the 34) CD patients after 12 months of gluten-free diet were then projected into the discriminant space of the model (stars) and were assigned to the CD or the healthy group applying a support vector machine [91] classifier (SVM). (b) Clustering of overt CD patients (open circles) and healthy controls (filled circles) obtained with CPMG serum spectra. The discriminant model was calculated using orthogonal partial least square [92] (OPLS) and validated using double cross-validation [93]. The CPMG spectra of 29 potential CD patients were then projected in the model (triangles) and filled or not according to the results of an SVM classifier. Adapted with permission from [49, 50]. Copyright (2009 and 2011) American Chemical Society.

The authors concluded that, although free from intestinal injury, placing potential CD subjects on a gluten-free diet could be justified because they are experiencing most of the pathological alterations experienced by overt coeliac patients [50].  Figure 3 shows the discrimination between overt CD patients and healthy controls and the statistical prediction of the potential CD patients: almost all potential CD patients are predicted as overt CD. The plot shown in Figure 3(a) was obtained using a training set composed of the serum CPMG spectra of 34 overt CD patients, 34 healthy controls, and 13 (out of the 34) CD patients after 12 months of gluten-free diet. It clearly appears that all but one patient on gluten-free diet were classified as healthy. Similarly, the plot in Figure 3(b) was built using the CPMG spectra of 61 overt CD patients, 51 healthy controls, and 29 potential CD patients. Almost all the potential CD patients fall in the CD group, underlining the affinity between the metabolic fingerprints of these two dissimilar clinical conditions.

Differences between the metabolic profiles of faeces and urine of CD and healthy children using a combination of ^1^H-NMR and GC-MS/SPME techniques were reported by di Cagno et al. [63]. The analysis allowed the identification of a group of compounds that were significantly changed in the treated coeliac children group. A set of volatile organic compounds and short fatty acids were identified using MS, whereas amino acids were identified using NMR [63]. Faecal and urine samples of treated CD children showed elevated levels of free amino acids (proline, methionine, histidine, and tryptophan) and lowered levels of some short fatty acids (butyric, isocaproic, and propanoic acids) compared to healthy children [63]. The authors suggested that these changes may be associated with intestinal and faecal bacteria modifications that could induce a nonspecific inflammation and a reduction of the absorptive surface of the intestinal mucosa; this may lead to a reduction of the absorption of amino acids which are subsequently lost with stool [63]. By combining microbiology and metabolomics, the authors showed that a gluten-free diet lasting at least two years did not completely restore the microbiota of the CD children. From that work, a broader picture seems to emerge that microbial indices (i.e., the ratio of faecal cell density of lactic acid *bacteria*-*Bifidobacterium* to *Bacteroides*-*Enterobacteria*) and the levels of some metabolites (i.e., ethyl-acetate, octyl-acetate, SCFA, and glutamine) are characteristic of CD patients [63].

## 6. Perspectives

Metabolomics is a rapidly growing discipline bringing together analytical technologies, metabolite pathways evaluation, and information technology. A major advantage is the noninvasive or minimally invasive measurement of potentially useful biomarkers from biofluids such as urine and plasma. A great deal of validation work (both at the analytical and data analysis level) has been carried out to gain full acceptance to metabolomics in routine clinical practice. Challenges for the development of metabolomics still exist, including simplified systems to present data to end-users (such as interpretation of often complex statistical models), the coordination of multiple data streams, and the implementation of quality control programs [94]. We expect that in the next few years it will be clear whether or not metabolomics will take its place as a complementary or even an alternative tool in the clinical setting.

At the present time, only few applications devoted to the investigation of coeliac disease have been presented in the literature, but a complex picture of the interaction between energy metabolism and gut microbiota seems to emerge, providing new hints on the biochemistry of the disease. In our institutions, as a logical complement to the results obtained analyzing overt coeliac and potential coeliac subjects, we are currently applying metabolomics to the biomolecular investigation of a gluten-related condition defined as gluten sensitivity [95]. This condition is still not very well characterized and its pathogenesis is caused by unknown mechanisms; we believe that metabolomics is a useful tool to expand our current limited knowledge of this condition.

Metabolomics-based approaches are expected to enable diagnosis, prognosis, and prediction of response of individuals to treatment. We can expect that metabolomics will provide more accurate and less expensive biomarkers (obtained by means of proper statistical analysis and properly validated) than presently available, which could improve diagnostic accuracy and sensitivity. However, far more research is essential to reach such a goal, and a validation of the results on an epidemiological scale is indeed needed.

**Box 1 (MS and metabolomics)**

The main analytical techniques used in metabolomics are nuclear magnetic resonance spectroscopy (NMR) and mass spectrometry (MS) [96]. Both MS and NMR methods provide information on a wide range of metabolites in a single measurement. Furthermore, both can be used to identify the metabolites' structures and to measure the relative and absolute concentrations of the molecules (MS has higher sensitivity but NMR is more reliable for determining concentrations) [97].

Mass spectrometry is a technique to determine extremely accurate mass of molecules in a pure sample or in a mixture. The molecules in a sample are converted to ions by an electron beam; the ions are accelerated by charged plates and then deflected by a magnetic field according to the mass-to-charge ratio of each ion. When the ions reach the detector, the mass-to-charge ratio is registered to provide a spectrum where series of peaks are shown reporting the intensity of each ion generated by the sample. MS is a destructive technique but requires a very low quantity of sample [98]. Over the last few years, its application to mammalian study increased, especially for its high sensitivity, and because it is a major technique for molecular identification [99]. As opposed to NMR, MS usually requires metabolites separation before detection, typically by using gas chromatography (GC) or liquid chromatography (LC). GC-MS is a robust technique for the analysis of volatile and semivolatile compounds suitable for chemical derivatization to increase their volatility [100]. Electron ionization (EI) in GC-MS is quite reproducible [100]. In contrast to GC-MS, LC-MS is especially suitable for the analysis of nonvolatile and/or thermally unstable metabolites. The introduction of UPLC (ultraperformance liquid chromatography) and capillary LC enabled better peak resolution and further increase in sensitivity and speed, and it is now successfully applied to metabolomics studies [101].

**Box 2 (NMR and metabolomics)**

NMR spectroscopy is an analytical technique that exploits the magnetic properties of certain atomic nuclei. It determines the physical and chemical properties of molecules by detecting the magnetically active nuclei. When placed in a magnetic field, an active nucleus (such as ^1^H or ^13^C) absorbs electromagnetic radiation at a characteristic frequency and then reemits it. After absorbing electromagnetic radiation in the range of frequencies of ^1^H (or ^13^C, or ^31^P,…), the sample emits all frequences of its active nuclei of that type, which constitute its ^1^H (or ^13^C or ^31^P,…) NMR spectrum. The resonance frequency and the corresponding intensity of each signal are dependent, respectively, on the chemical environment where that particular nucleus is located (i.e., molecular structure) and on the concentration of that molecule.

NMR spectroscopy is a nondestructive and highly reproducible technique and provides detailed information on the molecular structure of both pure compounds and complex mixtures [102]. In a typical biological fluid, all hydrogen-containing molecules will give a ^1^H-NMR spectrum as long as they are present in concentrations above the detection limit. The NMR spectrum of a biological fluid is therefore the superposition of the spectra of thousands of different small molecules (up to 2500 for urine and up to 200 for serum/plasma) present in the sample at concentrations >1 *μ*M [103]. An advantage of NMR is that the biological fluid requires only a mild treatment prior to the analysis.

The main disadvantage of NMR is its relatively low sensitivity. Another disadvantage of the NMR approach is the difficult identification of all metabolites in the samples: ^1^H-NMR spectra of biological fluids are very complex and often additional two-dimensional NMR experiments may be needed to assign metabolites in biofluids. The development of high-resolution ^1^H magic angle spinning (MAS) spectra made viable the acquisition of data on small slices of tissue without any pretreatment [104–106].

**Box 3 (biofluids and metabolomics)**

Most biofluids used in metabolomics can be collected noninvasively. The Human Metabolome Database (http://www.hmdb.ca/) lists 16 different biofluids investigated and up to 5000 identified or putative metabolites: amniotic fluid (17), aqueous humor (1), ascites fluid (1), bile fluid (18), blood (4297), breast milk (37), cellular cytoplasm (49), cerebrospinal fluid (436), faeces (0), lymph (1), menses (0), mucus (0), pericardial effusion (1), prostate tissue (13), saliva (70), sebum (0), semen (4), sweat (1), synovial fluid (0), tear fluid (1), urine (3873), and vaginal fluid (0). Of these, 694 have been associated with one or more diseases and pathologies.

Blood, urine, cerebrospinal fluid, and saliva are the richest in metabolites. The Human Serum Metabolome project. [107] (http://www.serummetabolome.ca/) lists 4229 *detectable* metabolites (most of them lipids) obtained by enhanced NMR, MS, and other analytical platforms. NMR was able to measure 1.2% (49/4229) of the human serum metabolome, GC 2.13% (90/4229), ESI-MS/MS (lipid mediator profiling) 2.3% (96/4229), and TLC/GC-FID-MS (general lipidomics) 79.9% (3381/4229, mostly, however, components of the complex lipid fraction) and DFI MS/MS is able to access 3.3% (139/4229) of the serum metabolome. Some of the compounds identified by NMR are urea (6 mM), glucose (5 mM), lactic acid, (1.4 mM), glutamine (0.51 mM), and glycerol (0.43 mM). The least abundant compounds were carnitine (46 *μ*M), acetic acid (42 *μ*M), creatine (37 *μ*M), cysteine (34 *μ*M), propylene glycol (22 *μ*M), and aspartic acid (21 *μ*M), and the lowest concentration reliably detected using NMR was 12.3 *μ*M (for malonic acid) and 14.5 *μ*M (for choline).

The Human Urine Metabolome project [108] (http://www.urinemetabolome.ca/) lists up to 3100 metabolites identified in urine. Human urine contains many classes of compounds excreted as waste products, including organic acids, amino acids, purines, pyrimidines, sugars, sugar alcohols, sugar acids, amines, and other compounds, at a variety of concentrations. Fresh urine is also characterized by the presence of human cells (erythrocytes, leucocytes, urothelial cells, and epithelial cells), bacteria, fungi, sperms, and noncellular components (mucus filaments, cylinders, cylindroids, pseudocylinders, and crystals, urates).

Some of the metabolites identified in saliva using NMR are [109] glucose, propionate, acetate, taurine, glycine, alanine, sucrose, dimethylamine, formate, glycine, lactate, methanol, propionate, propylene glycol, pyruvate, succinate, and taurine.

A large panel of metabolites has been also identified in cerebrospinal fluid by using NMR and GC-MS [36]. Among those obtained by NMR, there are amino acids, sugars, 2-oxoglutarate, 2-oxoisovalerate, 3-hydroxybutyrate, 3-hydroxyisovalerate, xanthine, and pyruvate.

Up to 50 metabolites were identified in faecal extracts via NMR [43]: amino acids, n-butyrate, propionate, n-caproate, 3-(4′-hydroxyphenyl) propionate, 5-aminopentanoate, glucose, 5-N-acetylneuraminate, 5-aminosalicylate, N-acetyl-5-aminosalicylate, deoxycholate, and phenylacetate, many of which are of bacterial origin.

**Box 4 (the metabolomics pipeline)**

The workflow of a metabolomics study is complex and each step has its own criticalities that need to be addressed. The metabolomics workflow can be summarized as follows [96, 110–112].

*Biological Question.* It includes definition of the biological/biomedical problem to be addressed. 

*Study Design.* It involves power analysis and treatment design.

*Data Acquisition.* It concerns quality control strategies, experimental setting (platform specific), Sampling, and measurement design.

*Data Preprocessing.* It is a fundamental step before analysis involving alignment, baseline correction (MR), phasing, alignment, bucketing (NMR), normalization, and scaling.

*Metabolite Identification.* It includes spectral matching (MS) and peak assignment (NMR). 

*Statistical Analysis.* It includes explorative (i.e., PCA and clustering), predictive (regression, PLS-DA), and univariate analysis and model optimization and validation.

*Biological Interpretation.* It involves embedding the results within the framework of existing biological knowledge.

**Box 5 (statistical analysis of metabolomic data)**

Metabolomic data are high dimensional in nature. Tens, hundreds, or even thousands of (un) identified metabolites (relative) concentrations are measured by means of NMR or MS platforms, usually on a limited number of samples. Biological information is retrieved from this data by means of univariate and multivariate statistical methods [27, 113, 114]. Multivariate methods make also use of covariances or correlations which reflect the extent of the relationships among the variables, in contrast to univariate methods that focus solely on the mean and variance of a *single* variable.

Commonly used univariate methods are *t*-test and ANOVA [115] together with their corresponding nonparametric versions [116] and with appropriate correction methods for multiple testing [117]. Multivariate methods are a broad category. When the interest centers on predicting or explaining one variable (either a group category like case/control or a continuous response) by the other variables, methods like multiple regression [118] or partial least squares regression and discriminant analysis (PLS-DA) [119] or its extensions like Multilevel PLS-DA [120], Orthogonal PLS-DA [121], and N-way PLS-DA [122] together with a proper optimization and validation of the models [93, 123, 124] are used. In other cases, the interest centers on providing insight into the underlying structure of the complete set of variables and other tools are used. Some examples are principal component analysis (PCA) [125], used to reduce the number of variables when there is correlation present and to explore relations between objects, or cluster analysis [62], used when objects have to be grouped to represent data structure. Hybrid methods like nearest shrunken centroids [126] or simplivariate methods [127] and machine-learning techniques like artificial neural networks [128], random forest [129], and support vector machines [91] are also used in metabolomics [27, 112, 130].

## Figures and Tables

**Table 1:** Most relevant findings, and associated references, for studies linking gut microbiota and CD.

References	Type of sample	Technique	Microbiota phylum/class	Relevant findings
Wacklin et al. (2013) [55]	Mucosa biopsy	PCR-DGGE (real-time polymerase chain reaction, denaturing gradient gel electrophoresis), 16S rRNA sequencing	* Firmicutes Bacteroides Proteobacteria Actinobacteria *	Diversity in mucosal microbiota of celiac disease patients is associated with the symptoms of the disease.
Nistal et al. (2012) [57]	Duodenal biopsies	PCR (polymerase chain reaction)	*Firmicutes Proteobacteria Bacteroidetes * *Actinobacteria * *Fusobacteria *	Composition of small intestinal microbiota is similar between adults and children; there is higher number of *Streptococcus* and *Prevotella* in healthy subjects.
Nadal et al. (2007) [58]	Duodenal biopsy	FISH (Fluorescent in situ hybridization), Flow cytometry detection.		In faeces and duodenum of CD children, smaller amount of harmless bacteria (*Lactobacillus* and *Bifidobacterium*) and higher number of harmful bacteria are found (*Bacteroides/Prevotella + E. coli*) compared to healthy children.
S$\overset{´}{\text{a}}$nchez et al. (2010) [59]	Duodenal biopsy	PCR-DGGE	*Bacteroidetes *	Reduced number of intestinal microbiota in CD children but also in treated CD children was noticed. Treatment with GFD does not restore the bacteria composition.
S$\overset{´}{\text{a}}$nchez et al. (2011) [60]	Faeces samples	PCR-DGGE		Studies were carried out on stools of infants with high/low risk of CD and different types of milk feeding. High-risk infants have higher prevalence of *Bacteroides vulgatus*, whereas low-risk infants have higher population of *B. uniformis, B. ovatus, *and* B*. *plebeius *considering the subgroup of either breast-fed or formula-fed infants.
Cheng et al. (2013) [61]	Duodenal biopsy	qRT-PCR (quantitative real-time PCR)	*Bacilli Bacteroides * *Clostridium * *Proteobacteria *	Overall microbiota composition in the duodenal mucosa is comparable between healthy and CD children, but studied groups differ regarding bacteria subpopulation profile.
Sellitto et al. (2012) [51]	Faeces samples	qPCR (quantitative PCR)	* Bacteroidetes* *Firmicutes *	Lack of microflora maturation during first 2 years of life in infants at risk of CD. Moreover, there was observed absence of *Bacteroidetes* and abundance of *Firmicutes*.
Sanz et al. (2007) [54]	Faeces samples	PCR-DGGE	*Actinobacteria* *Firmicutes *	*Lactobacillus* and *Weissella* are more abundant and diverse in treated CD patients and control subjects than in active CD individuals. Composition of lactic bacteria and *Bifidobacterium* differs between celiac children and age-matched controls.
Kaufman and Rousseeuw (2009) [62]	Intestine biopsies	PCR	*Proteobacteria *	There observed no statistical differences in bacteria composition between healthy and CD children. Nevertheless,*Haemophilus* was more common in CD patients and *Neisseria polysaccharea* in control individuals.
di Cagno et al. (2011) [63]	Faeces sample, duodenal biopsy	RAPD (random amplification of polymorphic DNA) -PCR	*Eubacteria *	Higher number of different *Eubacteria* classes was found in duodenum of coeliac children under gluten-free diet than in healthy children.
Medina et al. (2008) [64]	Faeces sample	PBMC (peripheral blood mononuclear cell) phenotyping and flow cytometric analyses	*Actinobacteria *	Studies regarding interaction between faecal bacteria and immune system response of coeliac disease patients. It appeared that Gram-positive bacteria such as *Lactobacillus *and *Bifidobacterium* may act as inhibitors of inflammation.
